# IgG Anti-ghrelin Immune Complexes Are Increased in Rheumatoid Arthritis Patients Under Biologic Therapy and Are Related to Clinical and Metabolic Markers

**DOI:** 10.3389/fendo.2019.00252

**Published:** 2019-04-18

**Authors:** Mildren Porchas-Quijada, Zyanya Reyes-Castillo, José Francisco Muñoz-Valle, Sergio Durán-Barragán, Virginia Aguilera-Cervantes, Antonio López-Espinoza, Mónica Vázquez-Del Mercado, Mónica Navarro-Meza, Patricia López-Uriarte

**Affiliations:** ^1^Instituto de Investigaciones en Comportamiento Alimentario y Nutrición, Centro Universitario del Sur, Universidad de Guadalajara, Ciudad Guzmán, Mexico; ^2^Instituto de Investigaciones en Ciencias Biomédicas, Centro Universitario de Ciencias de la Salud, Universidad de Guadalajara, Guadalajara, Mexico; ^3^Departamento de Reumatología, Clínica de Investigación en Reumatología y Obesidad, Guadalajara, Mexico; ^4^Instituto de Investigación en Reumatología y del Sistema Músculo Esquelético, Centro Universitario de Ciencias de la Salud, Universidad de Guadalajara, Guadalajara, Mexico

**Keywords:** ghrelin, rheumatoid arthritis, metabolic alterations, clinical activity, body composition

## Abstract

Rheumatoid arthritis (RA) is a systemic autoimmune disease associated with increased risk of cardiovascular disease and metabolic alterations. The mechanisms underlying these alterations remain unclear. Ghrelin is a gastrointestinal hormone with potent effects on food intake, body weight, metabolism, and immune response. Recent studies reported the presence of anti-ghrelin autoantibodies in healthy subjects and the levels and affinity of these autoantibodies were altered in anorectic and obese individuals. In this cross-sectional study we analyzed anti-ghrelin autoantibodies in RA patients and evaluated its relationship with clinical, body-composition and metabolic parameters. Clinical measurements of RA patients included the disease activity score-28 (DAS-28), inflammatory biomarkers, autoantibodies (RF and anti-CCP), body composition, glucose and lipid profile. Serum ghrelin levels were measured by enzyme-linked immunosorbent assay (ELISA). Free and total anti-ghrelin autoantibodies quantification (IgG and IgA isotypes) was performed by in-house ELISA. RA patients had lower IgG anti-ghrelin autoantibodies levels and higher immune complexes percentage (IgG+ghrelin) compared to the control group, while the IgA anti-ghrelin autoantibodies showed no significant differences. In the bivariate analysis, the percentage of IgG anti-ghrelin immune complexes positively correlated with BMI and ghrelin whereas in the multivariate regression model, the variables associated were DAS-28, body weight, visceral fat, LDL-C and TG (*R*^2^ = 0.72). The percentage of IgA anti-ghrelin immune complexes positively correlated with RF and anti-CCP and the multivariate regression model showed an association with RF and body fat percentage (*R*^2^ = 0.22). Our study shows an increased percentage of IgG anti-ghrelin immune complexes in RA patients despite ghrelin levels were similar in both groups, suggesting an increase in the affinity of these autoantibodies toward ghrelin. The associations found in the multiple regression analysis for anti-ghrelin immune complexes support the previously reported functions of these natural autoantibodies as carriers and modulators of the stability and physiological effect of the hormone. However, in RA both the disease activity and the RF appear to influence the formation of these anti-ghrelin immune complexes.

## Introduction

Rheumatoid arthritis (RA) is a systemic inflammatory autoimmune disease, associated with high incidence of cardiovascular disease (CVD) and metabolic alterations, reporting an increase of 50% in deaths related to CVD in RA patients compared to the general population (1). This elevated risk is not only related to the classical CV risks like hypertension and dyslipidemias, but also to the chronic systemic inflammation occurring in the patients (2, 3). In addition, the inflammatory status in RA is associated with modifications of body composition, specifically an increase of body fat mass and depletion of lean mass, known as rheumatoid cachexia that can be present simultaneously with obesity (4, 5). Both, cachexia and obesity have been associated with higher disability scores and elevated risk of progressive disability (6–9). It has been recognized that the treatment of RA patients with disease-modifying anti-rheumatic drugs (DMARDs) such as methotrexate (MTX) and biological drugs including TNF-α and IL-6 blockers, as well as co-stimulation inhibitors such abatacept exert beneficial effects on the metabolic profile (10). Nevertheless, the precise mechanisms underlying the body-composition and metabolic alterations in RA as well as its relationship with the pharmacological therapy remain poorly understood.

Research on appetite and metabolism regulating hormones has increased substantially in rheumatic autoimmune diseases such as RA during the last years. Accumulating data strongly indicate that these hormones also exhibit potent actions on the regulation of immune and inflammatory responses and may play a role both in the pathogenesis and development of comorbidities in RA (11–13). Ghrelin is a 28 amino acid gastrointestinal peptide with potent orexigenic effects as well as anti-inflammatory properties including the inhibition of cytokines such as TNF-α, IL-1β, and IL-6 produced by T lymphocytes and monocytes (14–17). It has been shown that ghrelin increases the expression of adhesion molecules and exerts anti-proliferative effects on microvascular endothelial cells (18, 19). Also, ghrelin has demonstrated regulatory effects on bone metabolism, as it promotes osteoblast differentiation and proliferation and inhibits apoptosis (20, 21). Nevertheless, the concentrations and clinical relevance of ghrelin in RA are still controversial considering that some investigations reported lower (15), higher (22) or even similar (23, 24) levels of this hormone in patients under DMARDs and/or biological therapy in comparison to healthy controls.

Recent studies described the presence of autoantibodies directed against ghrelin in healthy subjects, and altered levels and affinity of these natural autoantibodies were reported in anorectic and obese individuals sera; suggesting that anti-ghrelin autoantibodies, specifically immunoglobulins of the G isotype (IgG) may affect the hormone transport and function according to its affinity, as well as regulate its stability by protecting the hormone from degradation (25–28). Co-administration of ghrelin and ghrelin-reactive IgG extracted from plasma of *ob/ob* mice to male lean C57B16 mice, increased their daily food intake and induced a tendency to increase their body weight (28). This may indicate that the presence of these natural autoantibodies directed against ghrelin and other appetite-regulating peptide hormones are associated with metabolism and body weight alterations (29). However, the presence and the possible role of these autoantibodies in autoimmune diseases have not been addressed, especially in RA where the occurring metabolic and body-composition alterations are prominent and the underlying mechanisms remain barely explored. Therefore, in this study we analyzed serum samples of RA patients and controls to characterize the circulating anti-ghrelin autoantibodies of IgG and IgA isotypes and evaluate its relationship with metabolic profile, body-composition and clinical parameters in RA patients undergoing biological therapy.

## Materials and Methods

### Subjects

A cross-sectional study of RA patients and control subjects was performed. RA patients were previously classified according to the American College Rheumatism (ACR)/European League Against Rheumatism (EULAR) 2010 criteria (30). Those who had known history of other autoimmune, diabetes mellitus, renal, hepatic or CV disease, as well as those under 18 years of age or pregnant were excluded. Control subjects reported not having any diagnosed autoimmune, cardiovascular, hepatic, renal, infectious, or thyroid disease as well as not being under lipid-lowering medication. Patients were recruited from the Obesity and Rheumatology Research Center, located at Guadalajara, Jalisco, Mexico.

The study was approved by the Ethics Research Committee of University of Guadalajara (CEICUC, Review Board registry number CONBIOETICA14CEI03420150130) and was conducted according to the principles of the declaration of Helsinki. All participants were adults and voluntarily signed an informed consent before their inclusion in the study.

### Methods

#### Clinical Data

All patients and controls answered a demographic and clinical interview at the moment of the blood sample collection. Patients were evaluated by a rheumatologist that performed a general physical examination and assessed the number of painful and swollen joints for determining the RA clinical activity index by the disease activity score 28 (DAS-28) (31). The functional disability was assessed through the Health Assessment Questionnaire-Disability Index (HAQ-DI, Spanish version) (32). The erythrocyte sedimentation rate (ESR) was assessed through the Wintrobe method. C-reactive protein (CRP) and rheumatoid factor (RF) were measured by turbidimetric assays (Cat. No. COD31029 and COD31030A25, respectively, A25 Biosystems, Barcelona, Spain) using automatized equipment (BS-10; Mindray, Shenzhen, China). The cutoff value for RF positivity was 30 IU/mL, with a 95% specificity. Anti-cyclic citrullinated peptide antibodies (anti-CCP) were assessed using an ELISA kit (Cat. No. COD-FCCP600; Axis-Shield Diagnostics, Dundee, UK) following the instructions provided by the manufacturer; 5 U/mL were set as the cut-off point for positivity, with a 100 % specificity.

#### Body Composition and Anthropometry

All body-composition and anthropometric measurements were made by certified nutritionists. Body weight, musculoskeletal mass, body fat percentage (BF%) and visceral fat were measured by a bioelectrical impedance equipment (HBF-514C Omron Healthcare, Inc. Lakeside Drive Bannockburn, Illinois, USA) following the manufacturer instructions.

The participant's height was measured using a 2.05 m ± 1 mm scale portable stadiometer (Holtain Limited, Crynich, Difed, Britain Ltd. UK) following the technique described by Jellife and Jellife (33). The body mass index (BMI) was calculated by dividing weight in kg by the square of height in m. The waist circumference (WC) was measured midway between the lower rib and the iliac crest, at the end of a normal expiration to the nearest 0.1 cm.

#### Glucose and Lipid Profile

The blood samples were taken after an 8–12 h overnight fasting and centrifuged at 3,500 rpm during 15 min for serum separation. Samples were aliquoted and stored at −20°C until the day of the assay. Fasting glucose (Cat. No. 1001190), total cholesterol (TC, Cat. No. 41022), low-density lipoprotein cholesterol (LDL-C, Cat. No. BSIS51-E), high-density lipoprotein cholesterol (HDL-C, Cat. No. BSIS37-E) and triglycerides (TG, Cat. No.1001313) were determined by colorimetric enzymatic methods using commercial kits (all reagents by Spinreact, Girona, Spain). Dyslipidemias and impaired fasting glucose were defined according to the Adult Treatment Panel III (ATPIII) guidelines (34) as TC ≥240 mg/dL, LDL-C ≥160 mg/dL, TG ≥200 mg/dL, HDL-C <40 mg/dL, and glucose ≥110 mg/dL.

#### Quantification of Ghrelin and Anti-ghrelin Autoantibodies

The fasting total ghrelin concentrations were assessed using an ELISA kit (Cat. No. EZGRT-89K, Upstate Chemicon Linco, Millipore), according to the manufacturer's instructions. A subsample of the RA patients (*n* = 25) was used to assess the serum ghrelin levels since not all were fasting.

To measure the autoantibodies against ghrelin of both IgA and IgG isotypes, an in-house ELISA test was performed based on a published protocol (35). This test allows the quantification of free (autoantibodies unbound to ghrelin) and total (autoantibodies forming immune complexes with ghrelin plus the free form) using two types of sample dilution buffers that create normal (pH 7.4) or dissociative conditions (pH 8.9), respectively, giving information about relative autoantibodies levels and affinities. Slight modifications were made to the method after thorough standardization; including the RA patients and controls serum dilution (1:1,000), the time of serum incubation (2 h) on ELISA plate, and the detection antibody dilution (1:8,000 for IgG and 1:15,000 for IgA). Both detection antibodies were conjugated to horseradish peroxidase (Cat. No. MBS674609 and Cat. No. MBS176676 for anti-human IgG and IgA, both from MyBioSource, California, USA) and incubated for 2 h. Subsequently, the plates were washed 4 times and 100 μl of the substrate tetramethylbenzidine (TMB, Sigma Aldrich) were added to the plate and incubated for 15 min for color development. Finally, 50 μl of stop solution was added and the optical density (OD) was read at 450 nm on a plate spectrophotometer (Bio-Rad, California, USA). For each ELISA plate, 2 wells were set as blank and the mean OD values were subtracted from the mean OD values of samples. Blank OD values in ELISA tests were all below 0.1, indicating that there was no unspecific binding of the detection antibody. Patient and control samples were run in duplicate, obtaining values with a mean variation <5% between duplicates for both IgA and IgG. IgG and IgA anti-ghrelin autoantibodies serum levels were expressed as OD, whereas IgG- and IgA- ghrelin immune complexes percentage were calculated using a ratio between free and total immunoglobulins by the following formula:

$$\begin{array}{l} \text{Immune complexes percentage}=100-\left(\frac{Free\;OD}{\;Total\;OD}\;\;\right)\times 100 \end{array}$$

### Statistical Analyses

The data distribution was verified by D'Agostino-Pearson normality test and were reported as mean ± standard deviation (s.d.) for parametric data, and median (25–75th centiles) for variables with non-parametric distribution. Categorical variables were expressed as percentage and absolute frequency. Differences between two groups were assessed using Student's *t*-test or Mann-Whitney *U*-test for independent samples, according to data normality. To evaluate the relationship between clinical and metabolic variables with the anti-ghrelin autoantibodies a correlation analysis was carried out using Pearson's or Spearman's correlation tests in accordance with the data normality. Multivariate linear regression analysis was performed to analyze the association of clinical, biochemical and body-composition variables with the IgG and IgA anti-ghrelin immune complexes. Analyses were carried out using GraphPad Prism 6.0 (GraphPad Software, USA) and NCSS 2007 software (Number Cruncher Statistical System for Window, USA). The significance level was set at *p* ≤ 0.05.

## Results

### Patients Characteristics

Forty-nine RA patients and 32 control subjects were included in the study. The demographic, body composition, biochemical and clinical characteristics of both groups are shown in Table 1. The RA patients had a mean age of 50 ± 15 years, of which 87.7% were females. Controls had a mean age of 43 ± 8 years, of which 93.7% were females. The 30.6% of the RA patients reported past, and 8.2% current smoking habits while 9.4% of the control group reported past and 9.4% current smoking habits. Mean disease duration among patients was 8.5 ± 8.46 years, the ESR levels were higher in patients than in controls. The CRP levels in patients were found within normal values, whereas positivity for RF was 75 and 95% for anti-CCP. According to the DAS-28 score and the HAQ-DI questionnaire, on average, patients had moderate activity (3.43 ± 1.23) and low disability (0.54 ± 0.51). Most patients (91.8 %) were treated with politherapy, incorporating biological DMARDs (DMARDb).

**Table 1:** Demographic, body composition, biochemical, and clinical characteristics.

	**RA**	**Controls**	***p*-value**
**Characteristics**			
Demographics			
*n*	49	32	–
Age (years)	50 ± 15	43 ± 8	–
Female, % (*n*)	87.7 (43)	93.7 (30)	–
Smoking			–
Never, % (*n*)	61.2 (30)	81.2 (26)	–
Former, % (*n*)	30.6 (15)	9.4 (3)	–
Current, % (*n*)	8.2 (4)	9.4 (3)	–
Clinical Parameters			
RA duration (years)	8.50 ± 8.46	–	–
ESR (mm/h)	34.92 ± 13.93	27.53 ± 13.04	**0.021**
CRP (mg/dL)	3.40 (1.6–9.0)	–	–
RF (IU/mL)	80.05 (34.9–94.3)	–	–
Positives, % (n)	75.5 (37)	–	–
Anti-CCP (U/mL)	129.60 (35.1–412.8)	–	–
Positives, % (n)	95.9 (47)	–	–
DAS-28	3.43 ± 1.23	–	–
HAQ-DI	0.54 ± 0.51	–	–
Drug Treatment			
MTX + DMARDb^a^, %	71.4	–	–
MTX + Baricitinib, %	8.2	–	–
DMARDb^a^, %	20.4	–	–
Body Composition			
Weight	66.50 (57.3–71.8)	73.45 (63.3–79.6)	0.081
BMI (kg/m^2^)	26.69 ± 4.05	27.92 ± 5.26	0.245
WC (cm)	89.28 ± 11.69	89.13 ± 14.09	0.957
BF (%)	39.55 (33.8–44.4)	41.60 (33.5–45)	0.468
Visceral fat level	8.30 ± 3.10	11.90 ± 5.40	**0.000**
Biochemical Parameters			
TC (mg/dL)	198.5 (174.3–240)	214.5 (186–243)	0.794
HDL-C (mg/dL)	40.92 ± 16.82	48.09 ± 14.80	0.089
LDL-C (mg/dL)	113.50 (102.5–149.3)	141.50 (102.3–201.8)	**0.030**
TG (mg/dL)	123.50 (94.7–191.3)	143.50 (102.3–201.8)	0.403
Glucose (mg/dL)	87.75 (79.8–97.6)	93.25 (83.25–104)	0.137
Ghrelin (pg/mL)	636.20 (503–761.1)	642 (480.5–1063)	0.634

*Data are shown as mean ± s.d. or median (25–75th centile). TC, total cholesterol; HDL-C, high density lipoprotein cholesterol; LDL-C, low density lipoprotein cholesterol; TG, triglycerides; BMI, body mass index; WC, waist circumference; BF, body fat; ESR, erythrocyte sedimentation rate; RF, rheumatoid factor; Anti-CCP, anti-cyclic citrullinated peptide antibodies; CRP, C-reactive protein; DAS-28, disease activity score 28; HAQ-DI, health assessment questionnaire-disability index (Spanish version); MTX, methotrexate; DMARDb, biological disease-modifying anti-rheumatic drugs. Difference between groups was assessed by Student's t-test or Mann-Whitney U-test, p ≤ 0.05 values are highlighted in bold. ^a^ Including TNF, CD20, and IL-6 receptor inhibitors*.

The body-composition parameters were similar between RA patients and controls, except the visceral fat level which was higher (*p* < 0.01) among controls. On average, both groups were overweight (>25 kg/m^2^) (36) according to the BMI (26.69 ± 4.05 kg/m^2^ for RA patients and 27.92 ± 5.26 kg/m^2^ for controls). The WC mean value was 89 cm in both groups, indicating abdominal obesity according to the ATP III WC cutoff point for women (>88 cm) (37). Both groups showed an excess of body fat (>30%) (38). Alike, the lipid profile and glucose levels were similar between RA patients and controls being within the normality ranges, excluding the LDL-C median levels which were higher among controls (141.50 mg/dL) than patients (113.50 mg/dL). The median total ghrelin levels were 636.20 pg/mL for RA patients and 642 pg/mL for controls, showing no significant differences.

### Anti-ghrelin Autoantibodies Analysis

**Figure 1:**
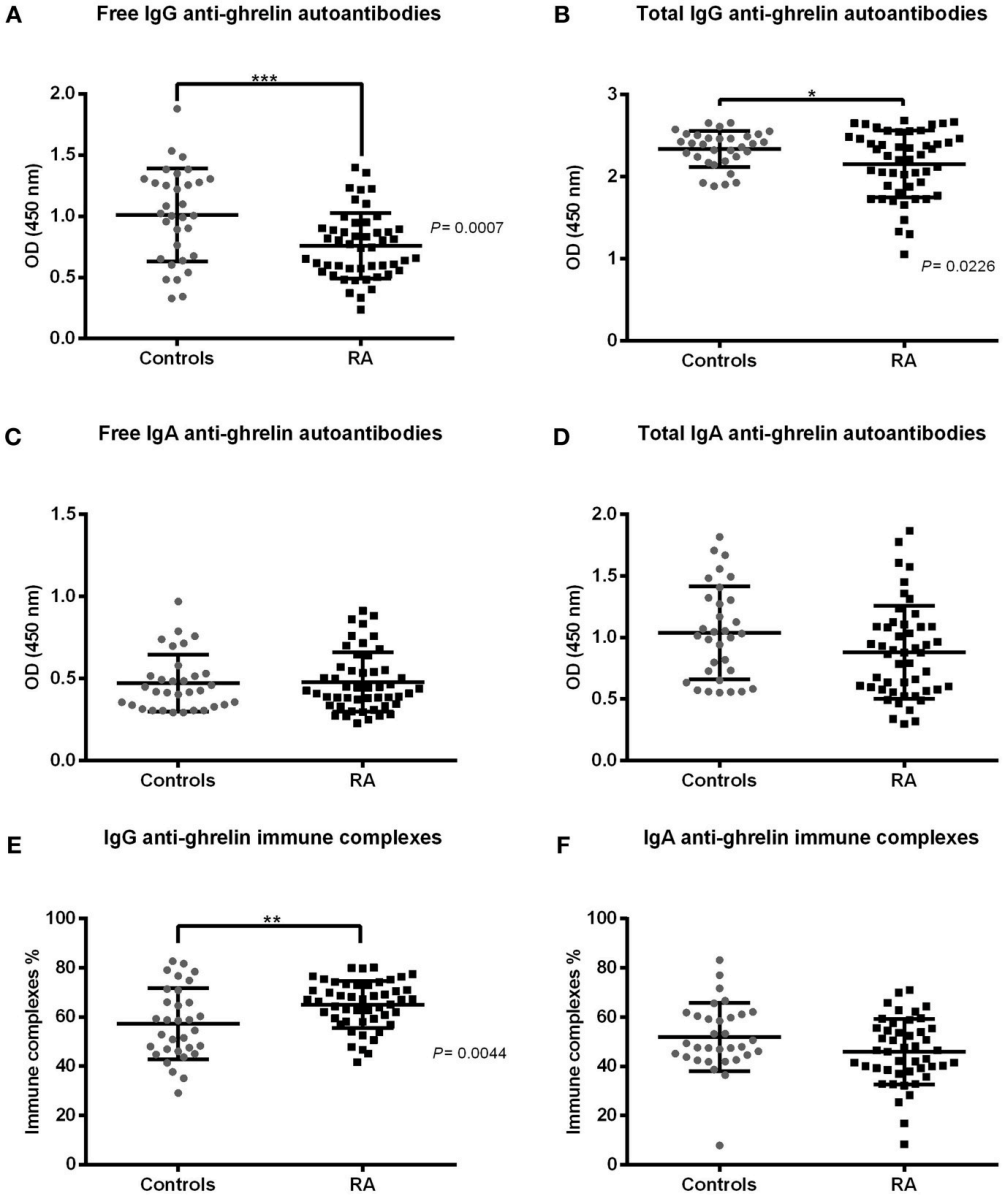
IgG and IgA anti-ghrelin autoantibodies levels in rheumatoid arthritis (RA) patients and controls. IgG and IgA anti-ghrelin autoantibodies serum levels are expressed in optical density (OD). **(A,C)** Free IgG and IgA autoantibodies. **(B,D)** Total IgG and IgA anti-ghrelin autoantibodies. **(E,F)** IgG and IgA immune complexes percentage. Controls n = 32, RA n = 49. Horizontal lines indicate mean and standard deviation. Difference between groups was assessed by Student's *t*-test or Mann-Whitney *U*-test, as appropriate. *P*-values ≤ 0.05 were considered statistically significant (*p < 0.05, **p < 0.01, ***p < 0.001).

Anti-ghrelin autoantibodies levels of both IgG and IgA isotypes are shown in Figure 1. Both free and total IgG anti-ghrelin autoantibodies were significantly higher in controls than in RA patients (*p* < 0.05) and on the contrary, the IgG immune complexes percentage was lower in controls than in RA patients (*p* < 0.05). While the IgA free and total anti-ghrelin autoantibodies levels, as well as the immune complexes percentage, showed no significant differences between controls and RA patients.

### Correlations Between Anti-ghrelin Autoantibodies With Clinical Parameters and Metabolic Profile in RA

We assessed the correlation between the clinical parameters in RA with the free and total fractions of IgG and IgA anti-ghrelin autoantibodies. Anti-CCP antibodies were positively correlated with total IgA anti-ghrelin autoantibodies (*r* = 0.326, *p* = 0.022) and the DAS-28 activity score showed a positive correlation with free IgA anti-ghrelin autoantibodies (*r* = 0.296, *p* = 0.050).When addressing the relationship between metabolic profile in RA with free and total fractions of anti-ghrelin, we detected a negative correlation between visceral fat level and total IgG anti-ghrelin autoantibodies (*r* = −0.519, *p* = 0.000). LDL-C levels were found positively correlated with free IgA anti-ghrelin autoantibodies (*r* = 0.404, *p* = 0.040). Ghrelin levels showed a negative correlation with free IgG anti-ghrelin autoantibodies (*r* = −0.534, *p* = 0.006).

**Figure 2:**
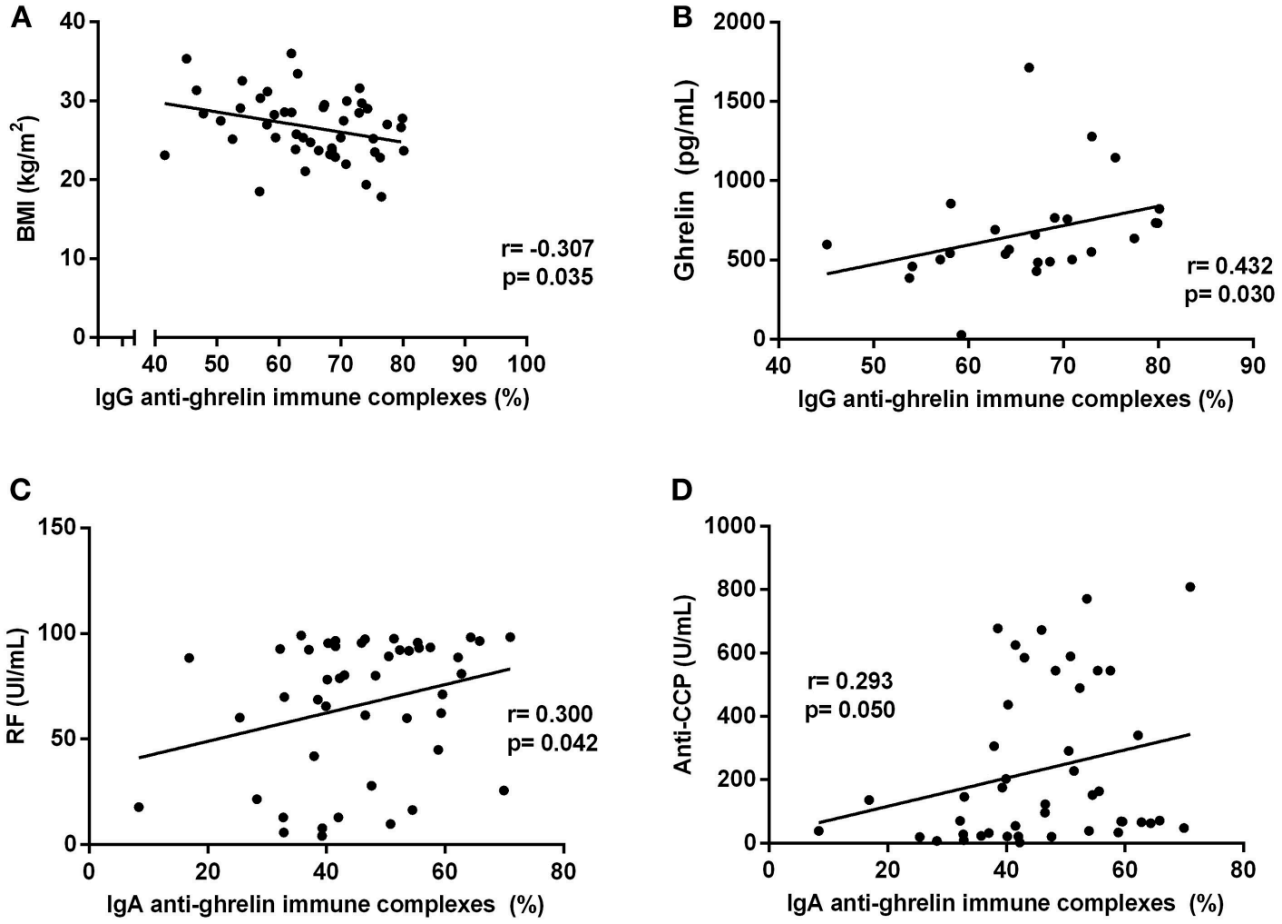
Correlations analysis of IgG and IgA anti-ghrelin immune complexes percentage with clinical and metabolic parameters in rheumatoid arthritis patients. **(A)** Correlation between IgG immune complexes % and BMI. **(B)** Correlation between IgG immune complexes % and ghrelin levels. **(C)** Correlation between IgA immune complexes % and RF. **(D)** Correlation between IgA immune complexes % and anti-CCP antibodies. BMI, body mass index; RF, rheumatoid factor; Anti-CCP, anti-cyclic citrullinated peptide antibodies. R, Spearman's or Pearson's coefficient, as appropriate. Statistical significance was considered at *p* ≤ 0.05.

The percentage of ghrelin-immune complexes also displayed significant correlations with both the clinical and metabolic parameters in RA (Figure 2); the RF and anti-CCP antibodies showed positive correlations with the IgA anti-ghrelin immune complexes percentage (*r* = 0.300, *p* = 0.042 and *r* = 0.372, *p* = 0.008, respectively) while the BMI was negatively correlated with IgG anti-ghrelin immune complexes percentage (*r* = −0.307, *p* = 0.035). Ghrelin levels were found positively correlated with the IgG anti-ghrelin immune complexes percentage (*r* = 0.432, *p* = 0.030).

### Multiple Regression Analyses of the Clinical, Biochemical, and Body-Composition Variables Associated With Anti-ghrelin Immune Complexes in RA

The IgG and IgA anti-ghrelin immune complexes percentage were further analyzed in a multiple regression model. We focused on the analysis of these complexes as they were significantly increased in RA patients and are likely implicated in the transport and modulation of biological effects of ghrelin (28). Thus, once we analyzed the simple correlations between the clinical, biochemical and body-composition parameters with both IgG and IgA anti-ghrelin autoantibodies levels, we selected the variables showing a significance of *p* < 0.20 as well as those previously reported to affect ghrelin concentrations.

The IgG anti-ghrelin immune complexes percentage was found associated with the DAS-28 score, weight, visceral fat level, LDL-C and TG concentrations explaining in a 72% its variance. While the IgA anti-ghrelin immune complexes were associated with RF and the BF percentage which explained a 22% of its variance (Table 2).

**Table 2:** Clinical, body composition, and biochemical parameters associated with IgG and IgA anti-ghrelin immune complexes in rheumatoid arthritis patients.

**Parameters**	**IgG immune complexes (%)^a^**		**IgA immune complexes (%)^b^**	
	**β**	***p***	**β**	***p***
DAS-28 (score)	−3.385	0.006		
Weight (kg)	−0.588	0.000		
Visceral fat	4.255	0.000		
LDL-C (mg/dL)	−0.121	0.003		
TG (mg/dL)	−0.086	0.003		
RF (IU/mL)			0.119	0.041
Body fat (%)			−0.503	0.028

DAS-28, disease activity score 28; RF, rheumatoid factor; LDL-C, low density lipoprotein cholesterol; TG, triglycerides. Multivariate analysis results from multiple linear regression analysis. The total explained variance of the model was

a *R^2^ = 0.72*,

b *R^2^ = 0.22*.

## Discussion

RA is associated with metabolic alterations, changes in body composition as well as increased risk of developing CV diseases (1, 2). Ghrelin, the main orexigenic hormone, is a gastrointestinal peptide with regulatory effects on metabolism and anti-inflammatory properties (15, 16). Recent studies reported the presence of natural autoantibodies directed against ghrelin in healthy individuals and altered levels and affinity of these autoantibodies in appetite-related pathologies including obesity and anorexia nervosa, suggesting a physiological role of these autoantibodies in ghrelin regulation (25, 27). In the present study, we evaluated for the first time the presence of anti-ghrelin autoantibodies of both IgG and IgA isotypes in RA patients under biological therapy and analyzed its relationship with body composition, metabolic profile and clinical activity parameters.

Analysis of ghrelin serum levels showed no significant differences in our cohort of RA patients in comparison to controls. Similarly to our findings, no significant differences in ghrelin levels were found among patients with established RA receiving traditional DMARDs treatment (17, 18) neither in patients under anti-TNF-α therapy (39) when compared to healthy controls. Conversely, a decrease in acyl-ghrelin levels in RA patients under DMARDs and/or biologics was reported in comparison to controls (15, 22), whereas higher levels of total ghrelin were observed after treatment with a TNF-α blocker in comparison to controls (22, 40). These apparently conflicting results may be explained by differences in the form of ghrelin measured in the studies (acyl, des-acyl, or total hormone levels), the patient's clinical characteristics as well as the treatment scheme.

Despite the lack of differences in ghrelin serum levels, we found that free and total IgG anti-ghrelin autoantibodies were decreased in our cohort of RA patients compared to controls. This decrease is probably an effect of the patient's immunosuppressive therapy as all of them were under MTX and/or biological therapy. MTX and abatacept have potent anti-inflammatory properties, and several clinical studies have demonstrated its effects on reducing serum immunoglobulins (41–44). Furthermore, MTX has demonstrated to lower total and transitional B cells as well as total T cell numbers in peripheral blood (41, 42, 45). Similarly, a decrement in both free and total IgG anti-ghrelin autoantibodies levels, as well as a decrease on their binding affinity with ghrelin, were observed in a rat model of methotrexate chemotherapy-induced anorexia (46). To support and extend the data obtained in this cross-sectional study it will be important to conduct longitudinal studies to further determine the effects of different RA treatment schemes on these natural anti-ghrelin autoantibodies.

In contrast to the decrease in free and total IgG anti-ghrelin autoantibodies in RA, we detected a higher percentage of IgG-ghrelin immune complexes in the patients as compared to controls. This is indicative of an increase on the affinity these autoantibodies toward ghrelin, probably as an adaptive mechanism in response to the reduction of the total fraction of anti-ghrelin autoantibodies among treated patients. We speculate that such affinity-mediated augment in the formation of anti-ghrelin immune complexes in RA, favor the stability and transport of ghrelin thereby enhancing its biological effects. Despite in the present study we did not measured the kinetic affinities of these autoantibodies in RA, previous studies performed by Fetissov and co-workers in other pathologies including obesity and anorexia nervosa can support our theory (28, 47). They demonstrated that ghrelin-reactive antibodies of the IgG isotype present a very variable Fab region showing distinctive affinities in the context of different pathologies (28). In obese individuals, increased kinetics affinities of IgG antibodies for the orexigenic hormone ghrelin were found and the transfer of these antibodies to mice promoted food intake and body weight gain. Similarly, IgG antibodies directed to the anorexigenic hormone leptin showed decreased affinity in obese humans (28, 48). It is worth mentioning that although there can be changes in the affinity of these ghrelin-reactive autoantibodies its affinity remains at the micromolar range and these can be classified as low-affinity, not being capable of neutralizing the hormone, but instead modulate its transport and/or protect it from degradation by serum enzymes (28). Even though the precise mechanisms fine-tuning the affinity of these natural autoantibodies remain unknown, these findings provide evidence that IgG anti-ghrelin autoantibodies undergo affinity modification in the context of metabolic pathologies as well as in immune-mediated diseases such as RA.

The IgG anti-ghrelin immune complexes percentage correlated with BMI and ghrelin levels in the bivariate analysis. This positive correlation with total ghrelin levels is reasonable, since it suggests that an increase in serum ghrelin levels cause more availability for the ghrelin-reactive antibodies to bound and form immune complexes. While in the multivariate regression model we found an interaction between DAS-28 score, body weight, visceral fat level, LDL-C, and TG concentrations, which explained a 72% of the variation in the percentage of these complexes. It should be noted that body weight, visceral fat level, LDL-C and TG have been previously correlated with ghrelin levels in several studies. The BMI and visceral fat mass were reported negatively correlated with plasma ghrelin (49, 50), which may indicate that the secretion of this hormone is reduced as a physiological adjustment in response to the positive energetic balance. In addition, it is recognized that ghrelin participates in lipid metabolism as it enhances adiposity by promoting the expression of several fat storage-related proteins in adipocytes (51). In concordance with our multivariate model, LDL-C and TG concentrations were reported negatively correlated with ghrelin in a study in obese children (52). In addition, Beaumont et al. (53) described that HDL-C particles may directly interact with ghrelin serving as a circulatory carrier, however, in our model HDL-C did not show an association with the percentage of ghrelin-immune complexes, suggesting that these antibodies are an independent carrier of the hormone.

The association of anti-ghrelin immune complexes with the DAS-28 in the multivariate analysis was striking, considering the fact that serum ghrelin levels alone were not associated with the disease activity nor with other clinical biomarkers in RA (data not shown); this suggests that anti-ghrelin autoantibodies but not ghrelin, are affected by the disease activity of the patients. Taken together, our results show that IgG-ghrelin immune complexes percentage exhibit the same associations previously reported for ghrelin and strongly support the idea of these autoantibodies as low-affinity carriers modulating the stability and biological effects of the hormone. However, in RA the IgG-ghrelin immune complexes formation appears to be negatively influenced by the disease activity. According to the multiple regression model it can be predicted that an increase on the disease activity as well as a rise in LDL-C and TG concentrations could affect the formation of IgG-ghrelin immune complexes. Based on the observation that patients in our study had an overall controlled RA (showing low to moderate activity according to DAS-28 score and low inflammatory biomarkers) this model may also explain the significant increase observed in the IgG anti-ghrelin immune complexes percentage in the patients, besides the previously discussed affinity-mediated compensatory mechanism that may promote ghrelin immune complexes formation.

While for the IgA anti-ghrelin autoantibodies levels, no differences were found between controls and patients. We hypothesize that this is related to the sample type used to quantify these isotype because IgA is not predominant in serum (in contrast to the IgG class), but is rather predominantly secreted in mucous membranes (54). Furthermore, the presence of IgA anti-ghrelin autoantibodies in serum, may indicate that these autoantibodies are triggered by antigens present in the intestinal lumen either under physiological or pathological conditions (27, 55). Since the gastrointestinal microbiota is a major physiological source of antigens, there has been suggested that bacterial epitopes can trigger cross-reactivity to regulatory peptides such as ghrelin, which displays sequence homology with commensal bacteria and viruses (27).

An interesting finding was the positive correlation observed between IgA anti-ghrelin immune complexes with RF and anti-CCP autoantibodies. The role of rheumatoid factors in the formation of immune complexes has been widely acknowledged in RA; this mechanism can enhance the inflammatory status through the release of inflammatory cytokines such as TNF-α and IL-6 via activation of Fc receptors expressed by innate immune cells such as macrophages. Nevertheless, this observation does not imply that anti-ghrelin complexes are considered pathogenic but rather regulatory, as these are detected in healthy individuals. In addition, autoantibody systems in RA are frequently correlated with each other; as described previously for anti-CCP and RF, and for RF and anti-PAD4 antibodies (56). Similarly, the multivariate regression model showed an association of the IgA anti-ghrelin immune complexes with RF and body fat percentage but only explained a 22 % of its variance. The negative association with body fat percentage again supports the hypothesis that the secretion of this hormone is reduced as a physiological adjustment in response to the positive energetic balance.

In summary, we evaluated for the first time the presence of IgG and IgA anti-ghrelin autoantibodies in RA patients and confirmed the previously reported presence of these natural autoantibodies in healthy individuals (26). Increased percentage of IgG anti-ghrelin immune complexes was found in RA patients under biological therapy with a low to moderate disease activity. This is indicative of an increase in the affinity of these autoantibodies toward ghrelin, probably as an adaptive mechanism in response to the reduction of the total fraction of anti-ghrelin autoantibodies among treated patients. In the multivariate regression analyses, the IgG-ghrelin immune complexes were associated with DAS-28, body weight, visceral fat, LDL-C, and TG while the IgA-ghrelin immune complexes were only associated to RF and body fat percentage, supporting the idea of these anti-ghrelin natural autoantibodies acting as carriers and modulators of the stability and physiological effect of the hormone. However, in RA both the disease activity and the hallmark autoantibody (RF) appear to influence the formation of these anti-ghrelin immune complexes. Future longitudinal studies should address the effects of different treatment schemes on these natural antibodies as well as monitor its changes in relation to clinical activity and metabolic changes in RA. In addition, analysis of autoantibodies directed to acyl-ghrelin or des-acyl ghrelin specific forms would be also informative.

## Ethics Statement

The study was approved by the Ethics Research Committee of University of Guadalajara (CEICUC, Review Board registry number CONBIOETICA14CEI03420150130) and was conducted according to the principles of the declaration of Helsinki. All participants were adults and voluntarily signed an informed consent before their inclusion in the study.

## Author Contributions

ZR-C was involved in the conception, design and performance of experiments, interpretation of data, and revision of the manuscript. MP-Q performed experiments, analyzed and interpreted the data, and wrote the article. JFM-V and SD-B aided in the design of the study, interpretation of data, and critical revision of the manuscript. MN-M provided help on data acquisition and interpretation. VA-C, AL-E, MV-D, and PL-U helped with the interpretation of data and critical revision of the paper. All authors read and approved the submitted version of the manuscript.

### Conflict of Interest Statement

The authors declare that the research was conducted in the absence of any commercial or financial relationships that could be construed as a potential conflict of interest.
